# Vancomycin presoak reduces infection in anterior cruciate ligament reconstruction: a systematic review and meta-analysis

**DOI:** 10.1186/s12891-023-06331-y

**Published:** 2023-04-05

**Authors:** Mingwei Hu, Yifan Zhang, Guangqian Shang, Jianjun Guo, Hao Xu, Xue Ma, Xue Yang, Shuai Xiang

**Affiliations:** 1 Department of Joint Surgery, The Affiliated Hospital of Qingdao University, No. 59, Haier Road, Laoshan District, Qingdao, Shandong China; 2 Operation Room, The Affiliated Hospital of Qingdao University, No. 59, Haier Road, Laoshan District, Qingdao, Shandong China

**Keywords:** Anterior cruciate ligament reconstruction, vancomycin, Infection, Antibiotic, Sports medicine

## Abstract

**Purpose:**

To compare the effect of vancomycin presoak treatment of grafts during anterior cruciate ligament reconstruction on the incidence of postoperative infection or septic arthritis.

**Methods:**

Studies published before May 3, 2022 investigating vancomycin presoak of grafts during anterior cruciate ligament reconstruction were searched in the PubMed and Cochrane Central Register of Controlled Trials. Studies were screened, and data on the incidence of postoperative infection or septic arthritis were extracted and included in the analysis.

**Results:**

Thirteen studies were included for analysis after search screening, yielding a total of 31,150 participants for analysis, of whom 11,437 received graft vancomycin presoak treatment, and 19,713 did not receive treatment. Participants who received vancomycin treatment had significantly lower infection rates (0.09% versus 0.74%; OR 0.17; 95% CI 0.10, 0.30; P < 0.00001).

**Conclusion:**

Pre-soaking of the graft with vancomycin during ACL reconstruction reduced the incidence of postoperative infection and septic arthritis.

**Supplementary Information:**

The online version contains supplementary material available at 10.1186/s12891-023-06331-y.

## Introduction

Anterior cruciate ligament reconstruction (ACLR) is an effective treatment for ACL rupture, ACLR can be reconstructed by single-bundle reconstruction, double-bundle reconstruction, or combined reconstruction. A meta-analysis conducted by Cheng et al. [1] also noted that combined reconstruction was found to be effective in improving rotational stability and leading to good functional scores. However, the choice of regimen should be individualized for the patient. Routine preoperative intravenous (IV) antibiotics to prevent infection have become standard for joint replacement or anterior cruciate ligament reconstruction. Although the incidence is low, several studies have shown that the postoperative infection rate of ACL reconstruction is 0.14-2.4% [2–10], and can lead to serious consequences. The prognosis for arthroscopic irrigation and debridement is currently good, but the outcomes appear to be poor compared to postoperative recovery in uninfected patients [11–14].

Vancomycin is a antibiotic effective against Gram-positive cocci, which acts by inhibiting the synthesis of the bacterial cell wall. This antibiotic exerts a strong effect against *Staphylococcus aureus, Streptococcus pyogenes, Streptococcus pneumoniae*, etc. Vancomycin has been widely used in the field of orthopedic surgery, to treat conditions such as septic arthritis. Topical application of vancomycin has also achieved good results in the non-orthopedic field, most notably in the field of orthopedic spine surgery, where topical application appears to be safe and effective [15]. In a prior randomized study, prophylactic intrawound administration of vancomycin reduced the risk of surgical site infection (SSI) during surgical fixation of fractures [16]. Vertullo et al. [17] first proposed in 2012 that the use of vancomycin presoaked tendon grafts could result in a significant reduction in postoperative infection rates. Furthermore, its hypoallergenic properties, thermal stability, topical safety, and large volume of distribution make vancomycin an ideal drug for the prevention of infection after arthroscopic anterior cruciate ligament reconstruction of the knee.

Therefore, the purpose of this meta-analysis was to compare the incidence of postoperative infection with vancomycin presoak grafts in ACLR with and without this treatment.

## Materials and methods

### Inclusion criteria

The subjects included in this study were adults who required anterior cruciate ligament reconstruction and received vancomycin-presoaked ACL grafts during anterior cruciate ligament reconstruction. The control group was patients who did not receive vancomycin-presoaked ACL grafts, regardless of the type of control received. The primary outcome was the incidence of infection after the intervention in the experimental group and the control group. The types of studies included randomized controlled trials (RCTs) and retrospective clinical trials.

### Search strategy

All studies were searched using the Preferred Reporting Items for Systematic Reviews and Meta-Analyses (PRISMA) guidelines. Two authors searched the medical databases PubMed and Cochrane Central Register of Controlled Trials. The search was carried out on May 3, 2022, and confirmed by discussion between the authors (MW.H, S.X). The search terms used were “(“anterior cruciate ligament“[MeSH Terms] OR (“anterior“[Title/Abstract] AND “cruciate“[Title/Abstract] AND “ligament“[Title/Abstract]) OR “ACL“[Title/Abstract]) AND (“vancomycin“[Title/Abstract] OR (“graft“[Title/Abstract] AND “soaking“[Title/Abstract]))”. All retrieved studies were included in the screening.

**Fig. 1:**
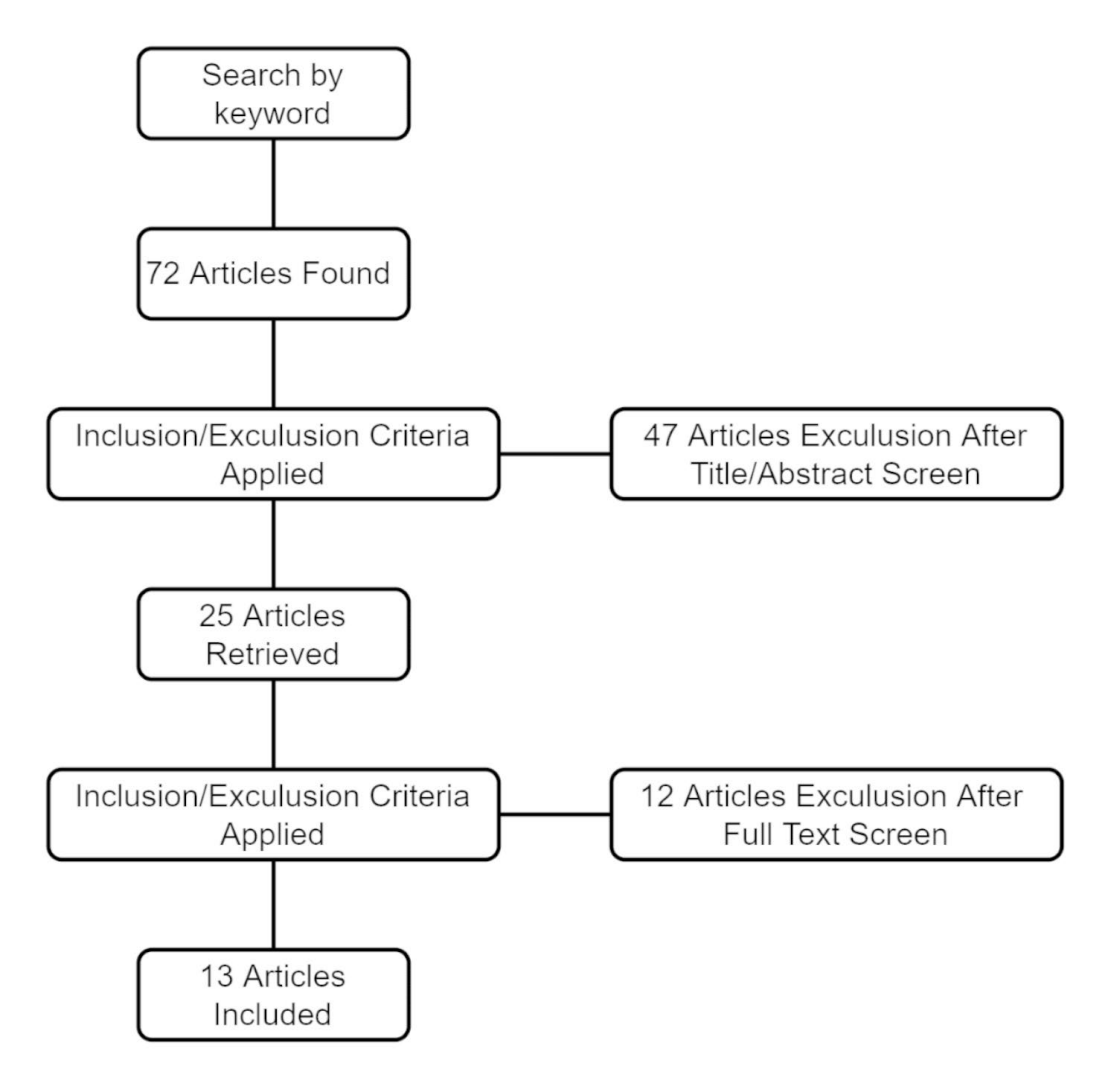
The selection process for inclusion and exclusion of articles

Eligible studies were those published in English which compared the outcomes of ACL reconstruction with vancomycin presoak and ACL reconstruction without vancomycin presoak. Infection rates and other relevant conditions were recorded in the study. Review articles, book chapters, and animal studies were excluded. Titles and/or abstracts were screened, and 2 authors independently evaluated them according to the inclusion and exclusion criteria to determine whether they were included in the study. Studies were thus identified for inclusion in the final analysis (Fig. 1).

Extracted data were cross-checked for accuracy by 2 authors, and recorded in a spreadsheet. Demographic parameters were collected for all studies and study subjects. Extracted study and subject demographic parameters included the year of study publication, duration of intervention, number of subjects, sex, age, diagnosis, graft type, preoperative antibiotic use, vancomycin concentration, and the preparation method of vancomycin presoaked grafts are prepared. All subjects were diagnosed with ACL rupture before and during surgery according to magnetic resonance imaging, physical examination, and arthroscopy. The outcome measure was postoperative infection rate.

The risk of bias in the included studies was analyzed using RevMan 5.4 (Fig S1-S2). The heterogeneity of postoperative infection rate outcomes was assessed using the I² index prior to meta-analysis. Results with an I²<40% were considered sufficiently homogeneous for meta-analysis [18]. The purpose of this analysis was to evaluate the effect of pre-impregnated and non-pre-impregnated ACL grafts with vancomycin on postoperative infection rates, and the measurement methods were sufficiently homogenized. The forest and funnel plots (Fig S3) in this analysis were constructed using RevMan 5.4.

We pooled the rates of postoperative infection of ACL grafts presoaked with vancomycin versus non-pre-impregnation. Because each study had a different time period, we performed a meta-analysis using a random-effects model and reported the data as having an Odds ratio (OR) with a 95% confidence interval (95% CI). All P values are reported with significance set at P < 0.05.

## Results

We initially obtained 78 articles by keyword search; after screening the abstract for eligibility, the full text of 25 was screened, yielding 13 articles for the final analysis [17, 19–30]. The defined evidence level of the articles was level III [20, 21, 23–29] or IV [17], although 3 articles did not mention the level of evidence [19, 22, 30]. A total of 31,150 patients were included in the study, of whom 11,437 received ACL graft vancomycin pre-soak and 19,713 did not receive ACL graft vancomycin presoak. Table 1 summarizes the general characteristics of the included studies, and Table 2 summarizes the data on postoperative infections in ACL grafts with and without vancomycin prep dip. All 13 studies involved autografts, and prophylactic antibiotics were given preoperatively. The antibiotics were cefazolin or other cephalosporins, and clindamycin or vancomycin was given intravenously for those with allergies. The concentration of prepreg solution was 1 mg/ml [20, 24], 2.5 mg/ml [22], and 5 mg/ml [17, 19, 21, 23, 25–30].

**Table 1:** Characteristics of the included studies

Study (Year of Publication)	Level of evidence	Mean age in vancomycin group (years)	Mean age in non-vancomycin group (years)	Number of patients in vancomycin group	Number of patient in non-vancomycin group	Placebo	Concentration of vancomycin	Time of soaked	Graft type	Tibial fixation	Femoral fixation
Banios 2021	Not mentioned	29.9	29.1	593	1242	Saline	5mg/ml	10 mintues	Hamstrings or bone–patella–tendon–bone	An absorbable interference screw	Endobutton or an absorbable interference screw
Baron 2019	III	27.9	27.6	798	842	Saline	1mg/ml	10 minutes	Autogratfs or allografts	Not mentioned	Not mentioned
Bohu 2020	III	30.3	29.9	490	1184	Saline	Not mentioned	N/A	Hamstring tendon, patellar tendon or the tensor fasciae latae tendon graft	Not mentioned	Not mentioned
Carrozzo 2022	III	28	29.3	2072	3228	Saline	2.5mg/ml	10 mintues	Bone–patellar ten- don–bone, quadriceps tendon, or HT autografts	Bioabsorbable screw, an interference screw and a cortical suspensory device	Bioabsorbable screw, an interference screw and a cortical suspensory device
Figueroa 2019	III	Not reported (mean age of all sample 29.1)	Not reported (mean age of all sample 29.1)	260	230	Saline	5mg/ml	15 minutes	Hamstrings	A BioComposite interference screw	A cortical button
Hees 2021	III	31.4	32.9	536	636	Saline	1mg/ml	N/A	Autologous hamstring tendons	An interference screw and an Endotack® (Karl Storz, Tuttlingen, Germany)	Flipp tack® (Karl Storz, Tuttlingen, Germany)
Offerhaus 2019	III	31.2	32.4	853	926	Saline	5mg/ml	N/A	Hamstrings, bone-patella- tendon-bone and quadriceps tendon	Absorbable interference screw	Endobutton and absorbable interference screw
Perez-Prieto 2016	III	Not reported	Not reported	734	810	Saline	5mg/ml	10–15 mintues	Quadrupled hamstrings and a BPTB	A resorbable interference screw	A resorbable interference screw or a transversal fixation or with a cortical suspensory fixation system
Phegan 2016	III	29	30	1300	285	Saline	5mg/ml	N/A	Hamstring graft	RCI screw and staple (Smith and Nephew Endoscopy)	Endobutton CL (Smith and Nephew Endoscopy, Andover, MA)
Schuster 2020*	III	Not reported	Not reported	2294	8222	Saline	5mg/ml	N/A	Hamstring tendon or quadriceps tendon	A hybrid fixation with a small interference screw above the epiphysial plate and non-absorbable suture material fixed to a screw or washer	A cortical fixation
Schuster 2020**	III	Not reported	Not reported	1638	517	Saline	5mg/ml	N/A	Hamstring tendon or quadriceps tendon	a hybrid fixation with interference screw (biodegradable or PEEK) and non-absorbable sutures fixed to a cortical button or screw	Aperture fixation with interference screws
Vertullo 2012	IV	31	30	870	285	Saline	5mg/ml	N/A	Hamstring graft	RCI screw and staple (Smith and Nephew Endoscopy)	Endobutton CL (Smith and Nephew Endoscopy, Andover, MA)
Wan 2020	Not mentioned	26.6	27.9	120	185	Saline	5mg/ml	15–20 minutes	Hamstring graft	A resorbable interference screw (BioRCI-Smith and Nephew)	A suspensory fixation system (EndobuttonTM-Smith and Nephew)

**Fig. 2:**
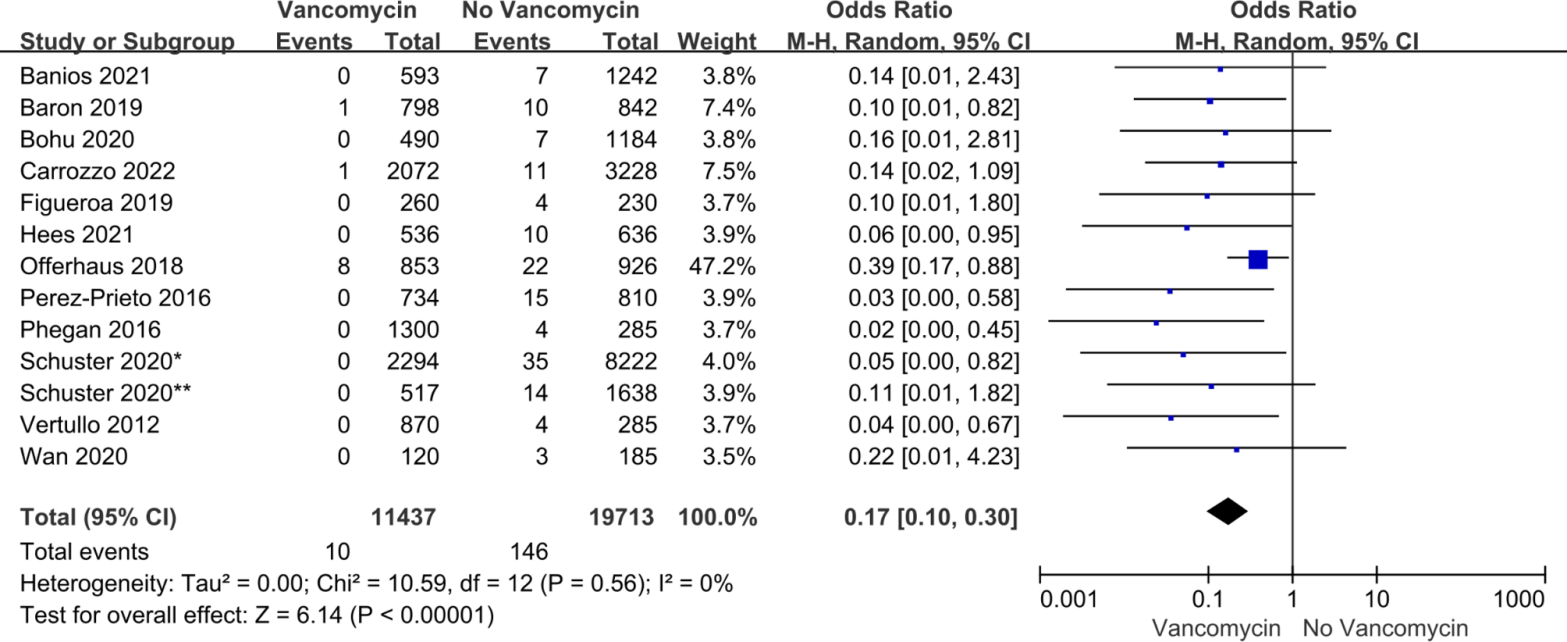
Forest plot of postoperative infection differences between ACL grafts soaked in vancomycin, and grafts not treated with vancomycin

All 13 studies reported on postoperative infections and included a total of 31,150 participants, of whom 11,437 received vancomycin presoak and 19,713 did not receive vancomycin treatment. Infection rates were significantly lower in those receiving grafts with vancomycin treatment (0.09% versus 0.74%; OR 0.17; 95% CI 0.10, 0.30; P < 0.00001). (Fig. 2)

**Table 2:** Date regarding infection after anterior cruciate ligament reconstructions with and without Vancomycin presoaked grafts

Study	Infected patients with vancomycin presoaked grafts/total patients (%)	Infected patients with non-vancomycin presoaked grafts/total patients (%)
Banios 2021	0/593 (0)	7/1242 (0.6%)
Baron 2019	1/798 (0.1%)	10/842 (1.2%)
Bohu 2020	0/490 (0)	7/1184 (0.6%)
Carrozzo 2022	1/2072 (0)	11/3228 (0.3%)
Figueroa 2019	0/260 (0)	4/230 (1.7%)
Hees 2021	0/536 (0)	10/636 (1.6%)
Offerhaus 2019	8/853 (0.9%)	22/926 (2.4%)
Perez-Prieto 2016	0/734 (0)	15/810 (1.9%)
Phegan 2016	0/1300 (0)	4/285 (1.4%)
Schuster 2020*	0/2294 (0)	35/8222 (0.4%)
Schuster 2020**	0/517 (0)	14/1638 (0.9%)
Vertullo 2012	0/870 (0)	4/285 (1.4%)
Wan 2020	0/120 (0)	3/185 (1.6%)

## Discussion

This meta-analysis revealed that presoaking of grafts for anterior cruciate ligament reconstruction with vancomycin significantly reduces the incidence of postoperative infection and septic arthritis.

Postoperative infection and septic arthritis are devastating complications which can occur after joint surgery, commonly caused by the pathogens *Staphylococcus aureus* or coagulase-negative staphylococci. Vancomycin exerts an antimicrobial activity against a wide range of gram-positive cocci and is widely used in orthopedic surgery, especially joint surgery, and has become one of the prophylactic antibiotics of choice for joint replacement surgery, ligament reconstruction, etc. The use of vancomycin for the presoaking of ligament grafts to reduce the incidence of postoperative infection and septic arthritis was first proposed by Vertullo et al. [17]. Vancomycin can further be used as a remedy after contamination has occurred in ligament reconstruction surgery. Perez-Prieto et al. [31] showed that ACL graft harvesting and manipulation lead to bacterial contamination in 14% of cases. This contamination can be fully eradicated by soaking in vancomycin solution.

Grayson et al. [32] described the in vitro elution properties of vancomycin from the tendon in 2011. The amount of drug released from the tendon by vancomycin and the elution profile depends on the washout, the size of the tendon, and the concentration of the vancomycin-infused solution used in the protocol. Vancomycin elution does not last long, and therefore should not pose a risk to osteoclasts and chondrocytes due to local accumulation of the drug. Care should be taken when flushing the tendon, as vancomycin concentrations increase immediately when the tendon is not flushed. Further in vitro and in vivo studies are needed to determine local vancomycin concentrations after insertion of tendons prepared using this protocol, and to assess the clinical benefits and risks of using vancomycin in this procedure. Vancomycin intervention on primary human tendon cells in vitro is safe at a concentration of 2.5 mg/ml for up to 60 min, whereas the higher concentrations commonly used during vancomycin wrapping may impair graft tendon cell activity and metabolic activity [33]. However, another study showed that human knee tendon-derived tendon cells do not exhibit significant cell death and toxicity after short-term exposure to relatively high concentrations of vancomycin. However, in the presence of high vancomycin concentrations, tendon cell morphology changes from the typical elongated spindle shape to a rounded shape [34]. The vancomycin treatment time in the included studies was approximately 10–15 min, and the balance between infection prophylaxis and cytotoxicity is still a trade-off for clinical application. A meta-analysis conducted by Naendrup et al. [35] concurrently noted that soaked of grafts in vancomycin solution for at least 20 min resulted in complete removal of bacteria and that no differences in tendon biomechanical properties were detected in subsequent tests. In the rat model, Tong et al. [36] performed ACLR with grafts soaked in vancomycin and performed biomechanical tests after surgery to show that vancomycin soaked could effectively prevent Staphylococcus aureus contamination and did not affect tendon-bone integration and knee function, so as to verify the effect and influence of vancomycin soaked in animals. We hope that more studies will explore this in depth.

Vancomycin has been proved to be chondrotoxic in vitro, which is proportional to the concentration of vancomycin. The concentration of vancomycin used in the joint should be strictly controlled [37]. Shaw et al. [38] ‘s study showed that vancomycin was toxic to articular chondrocytes at a concentration of 5 mg/ml or higher. A strategy to control the concentration of vancomycin should be developed before the application of topical vancomycin around the synovial joint, so as to prevent the occurrence of cartilage and joint diseases caused by vancomycin soaked in cartilage and osteochondral tendon grafts [38]. Soaked of tendon grafts with vancomycin resulted in sustained release and higher concentrations than the minimum inhibitory concentration of staphylococci, and vancomycin elution was lower than previously reported toxic concentrations for osteoblasts and chondrocytes.

Postoperative joint infections are devastating complications that are difficult to treat, and the costs associated with treatment and care are a huge financial strain on patients. By analyzing the baseline postoperative infection rates in the literature and the costs of antibiotics and infection-related care, Ruelos et al. [39] used a break-even economic analysis to demonstrate that the application of vancomycin implants or prepreg in arthroscopic ACL reconstruction is an effective, economical method to prevent infection effective prophylactic measure which can greatly reduce the financial stress of patients. In addition, the 2022 study by Truong et al., [40] also showed that the vancomycin presoake technique is an extremely cost-effective way to prevent septic arthritis after ACLR, but also noted that if the infection rate is less than 0.014% when intravenous antibiotics are used, there is no significant difference in the incidence of septic arthritis after ACLR. The vancomycin presoak technique would no longer be cost-effective. Vancomycin presoak technique should be taken into account when performing surgery and choosing postoperative anti-infective treatment. The use of vancomycin in arthroscopic ACL reconstruction should thus be encouraged and advocated.

In terms of re-rupture rates, Perez-Prieto et al. [41] performed a retrospective study with a minimum 5-year follow-up period and found that the vancomycin-infusion technique for autologous ACL grafts appeared to be a safe approach as far as re-rupture rates were concerned, and did not compromise the functional outcome after ALC reconstruction. In all of the studies we included in our analysis, autologous tissue was used as the graft for ACL reconstruction. For ACL reconstructions performed with autologous grafts, the use of vancomycin-impregnated grafts to prevent infection has been concluded to be significantly effective, and can thus be an effective measure to prevent infection after ACL reconstruction.

Despite the promise of this treatment method, our study still has many limitations. First, despite the large sample size of clinical studies, the follow-up period is still short, and infection after cruciate ligament reconstruction is an evaluation index that requires long-term monitoring. Further, more studies with high-quality medium- and long-term follow-up clinical outcomes are still needed to support its validity. Second, most of the studies included in this analysis were retrospective studies with a low level of evidence, and thus, prospective randomized controlled trials with large sample sizes are required to provide better guidance on clinical treatment. Third, the definitions of postoperative infection and septic arthritis were different in many of the included studies, and the inclusion and exclusion criteria were not elaborated. In addition, some studies mentioned the evaluation of postoperative functional and clinical outcomes of patients after vancomycin-infused grafts, but some of these evaluations only used subjective evaluations such as telephone follow-up, without applying objective evaluation indicators, and more data are needed to assess the immediate and long-term outcomes of postoperative clinical outcomes after vancomycin-infused grafts in this regard. Finally, all the studies included in this analysis were autogenous tissue tendon grafts, and the reaction of synthetic grafts such as artificial materials such as LARS ligament to vancomycin soaked was not discussed, and their biomechanical properties and risk of re-rupture still need to be clarified and discussed.

## Conclusion

This meta-analysis revealed that pre-soaking of grafts with vancomycin during ACL reconstruction reduced the incidence of postoperative infection and septic arthritis.

## Electronic supplementary material

Below is the link to the electronic supplementary material.

Supplementary Material 1

Supplementary Material 2

Supplementary Material 3

Supplementary Material 4

Supplementary Material 5

## Data Availability

All data generated or analysed during this study are included in this published article [and its supplementary information files].
