# Supplementary figures and images for: Vancomycin presoak reduces infection in anterior cruciate ligament reconstruction: a systematic review and meta-analysis
Source: BMC Musculoskelet Disord. 2023 Apr 5;24:267. doi: 10.1186/s12891-023-06331-y (PMC10074692; doi:10.1186/s12891-023-06331-y)

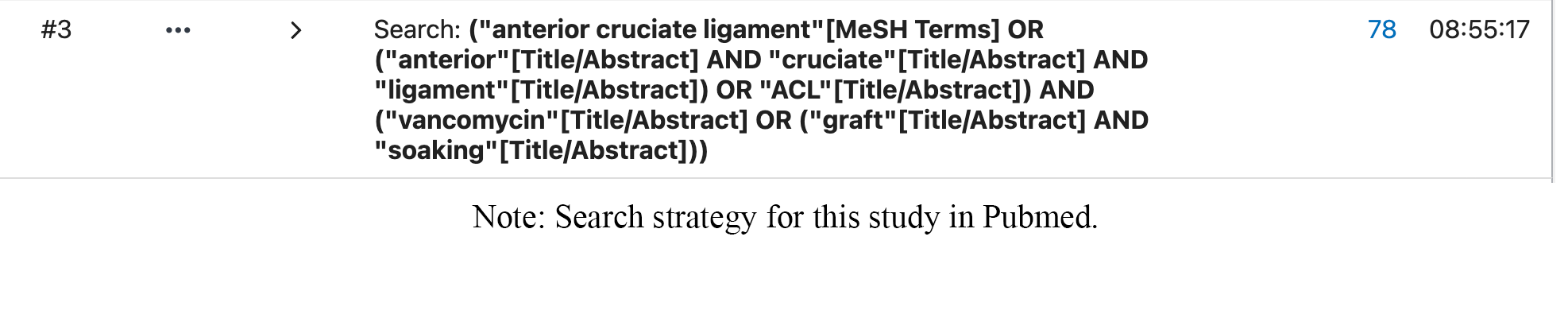

Supplement: Supplementary file 1 — Supplementary Material 1 [file 12891_2023_6331_MOESM1_ESM.tif]

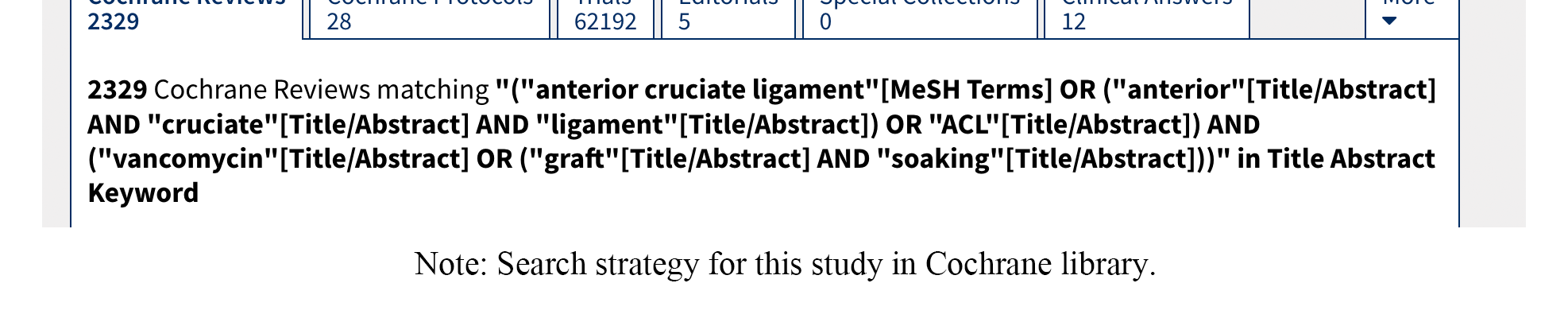

Supplement: Supplementary file 2 — Supplementary Material 2 [file 12891_2023_6331_MOESM2_ESM.tif]

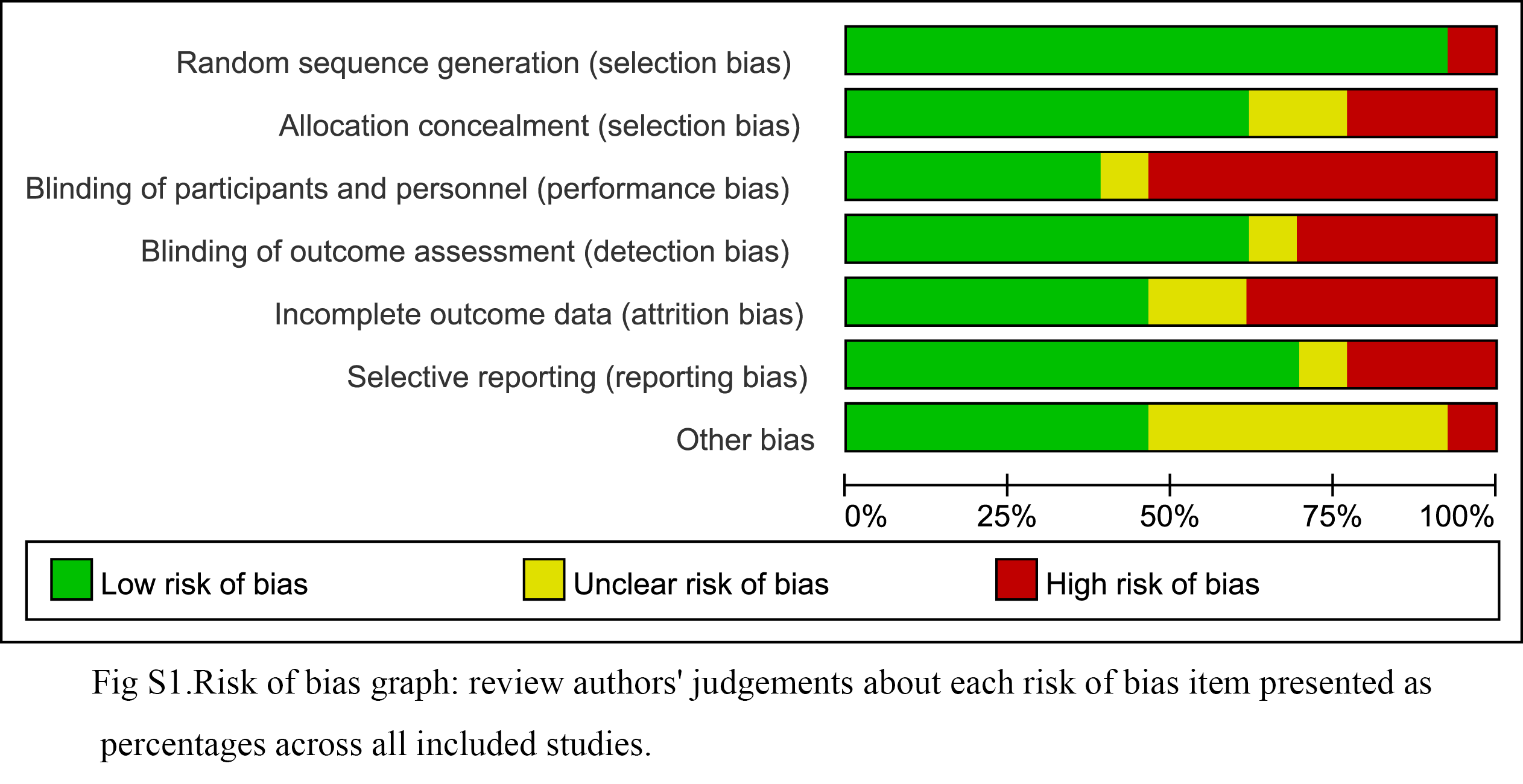

Supplement: Supplementary file 3 — Supplementary Material 3 [file 12891_2023_6331_MOESM3_ESM.tif]

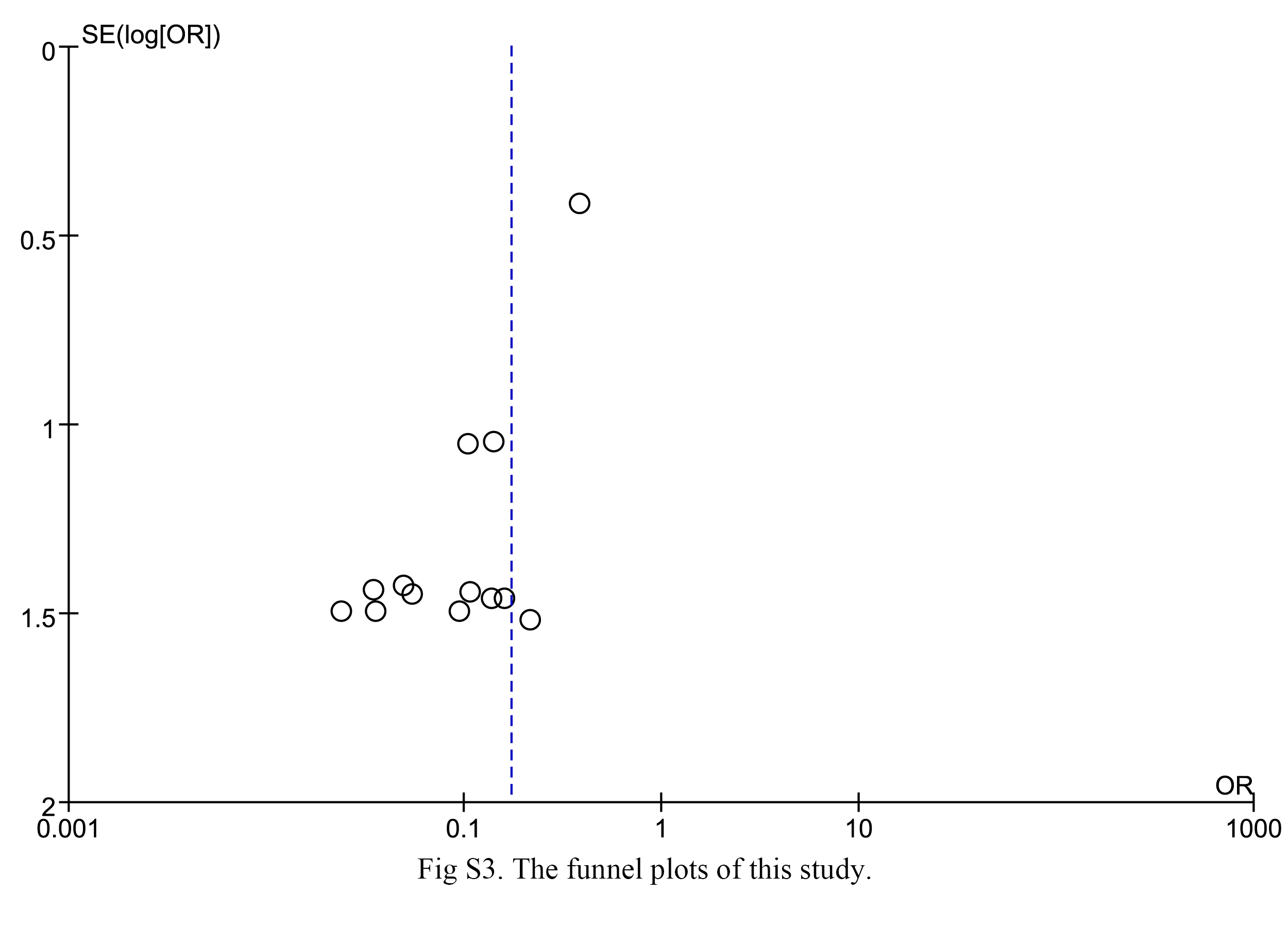

Supplement: Supplementary file 4 — Supplementary Material 4 [file 12891_2023_6331_MOESM4_ESM.tif]

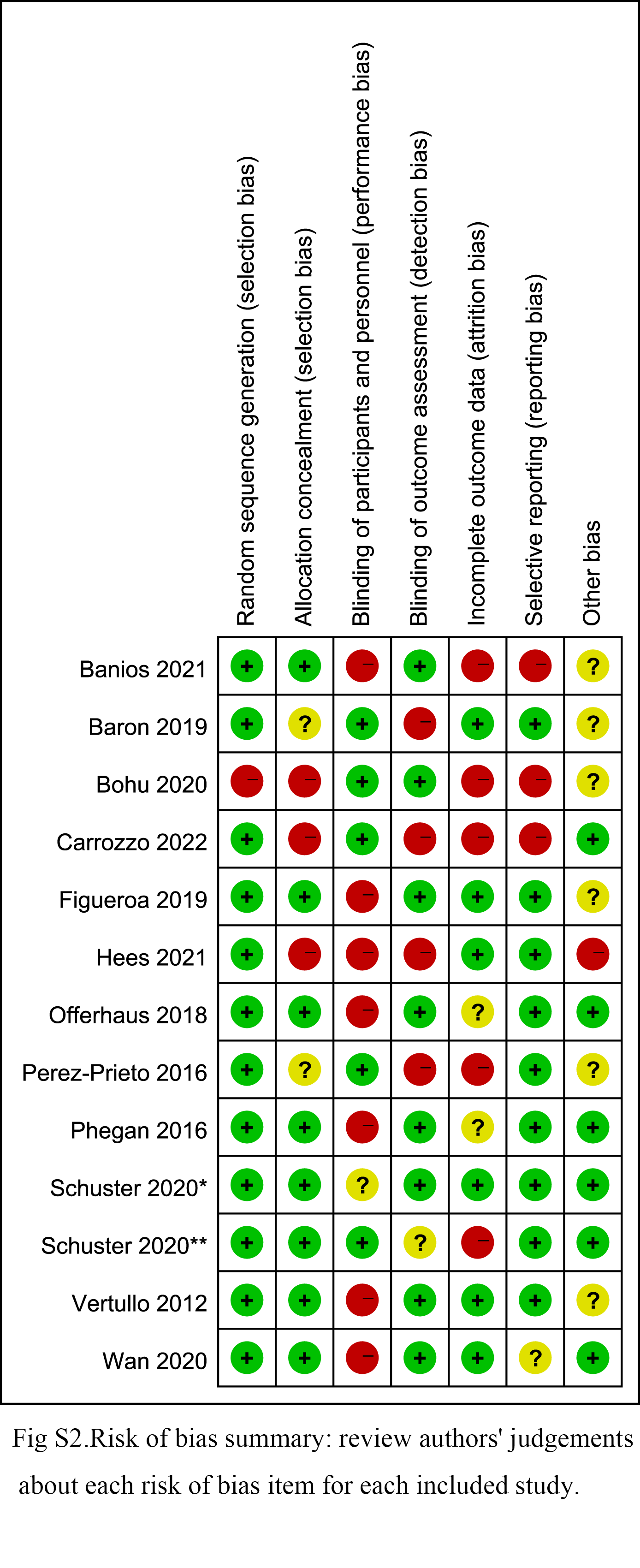

Supplement: Supplementary file 5 — Supplementary Material 5 [file 12891_2023_6331_MOESM5_ESM.tif]
